# Reimagining research equity and beneficial community impact: reflections from the GSK Africa Open Lab inaugural cohort

**DOI:** 10.1080/16549716.2026.2718934

**Published:** 2026-08-24

**Authors:** Adwoa Kumiwa Asare Afrane, Belinda L. Lartey, Adebanjo Adegbola, Almahamoudou Mahamar, Ernest Adankwah, Kelvin Kering, Jabar B. P. Agbo Achimi Abdul, Josephine Tumuhamye, Stephen Tukwasibwe, Victoria Etuk, Ousmane Traore

**Affiliations:** a Department of Child Health, University of Ghana Medical School & Korle Bu Teaching Hospital, Accra, Ghana; b Department of Electron Microscopy and Histopathology, Noguchi Memorial Institute for Medical Research, University of Ghana, Legon, Ghana; c Faculty of Pharmacy, Obafemi Awolowo University, Ile-Ife, Nigeria; d Malaria Research and Training Center, University of Science, Techniques and Technology of Bamako, Bamako, Mali; e Department of Medical Diagnostics, Faculty of Allied Health Sciences, Kwame Nkrumah University of Science and Technology, Kumasi, Ghana; f Centre for Microbiology Research, Kenya Medical Research Institute, Nairobi, Kenya; g Centre de Recherches Médicales de Lambaréné CERMEL, Hospital Albert Schweitzer, Lambaréné, Gabon; h School of Public Health, Makerere University, Kampala, Uganda; i Infectious Diseases Research Collaboration (IDRC) and Department of Medical Biochemistry, Makerere University, Kampala, Uganda; j International Research Centre of Excellence, Institute of Human Virology, Abuja, Nigeria; k Institut de Recherche en Sciences de la Santé, Clinical Research Unit of Nanoro (CRUN), Nanoro, Burkina Faso

**Keywords:** Infectious diseases, research capacity strengthening, research equity, scientific mentorship, sub-Saharan Africa

## Abstract

Global health research continues to rely heavily on knowledge generated outside the regions that bear the greatest disease burden, reinforcing structural inequities in scientific leadership and innovation. The GlaxoSmithKline (GSK) Africa Open Lab (AOL) was established to address this imbalance by investing directly in African-led research capacity. This reflective analysis examined how participation in the inaugural GSL-AOL cohort influenced research capacity, scientific independence, and career development among early-career African researchers. The reflection was guided by the question: *How has your participation as part of the inaugural cohort of the GSK AOL programme influenced your research capacity, scientific independence, and career trajectories as an early-career African researchers?* A structured collaborative reflection approach was undertaken involving all 11 members of the inaugural cohort. Reflections were analyzed using Ritchie and Spencer’s Framework Method, with responses independently reviewed, coded, and synthesized into consensus themes. Drawing on our collective experiences and potential outputs, we reveal how catalytic funding, structured mentorship, and intentional network-building can accelerate research independence, strengthen institutional capacity, and expand professional opportunities. The cohort’s achievements provide evidence that African-led, researcher-centered investment models can contribute meaningfully to equitable global health research. Sustained impact, however, will require continued support from African governments, institutions, and international partners.

## Paper Context

- **Main findings:** This paper examines the impact of the GSK Africa Open Lab on its first cohort of researchers. It demonstrates that supporting young African scientists with funding, mentorship, and networks helps them become independent research leaders, strengthens local laboratories, and produces critical evidence for fighting diseases in Africa. Importantly, it shows that relatively modest, well-planned investments can lead to significant scientific and policy impact.
- **Added knowledge:** While global health research frequently highlights leadership imbalances, practical solutions remain underexplored. This paper addresses this gap by examining a capacity-strengthening programme that supports African-led research. Drawing on participant experiences, it identifies four key factors that help build robust local research systems: giving researchers grant ownership, providing quality mentorship, collaboration, and ensuring responsible data management.
- **Global health impact for policy and action:** Strengthening research in low- and middle-income countries takes time and commitment. The GSK Africa Open Lab shows one of the ways forward; a model where different partners work together to support African-led science. For this to last, everyone needs to play their part. Countries need to invest more in their own research, and international partners must stay engaged for the long haul.

## Background

### Addressing structural inequities in global health research

**Figure 1.**
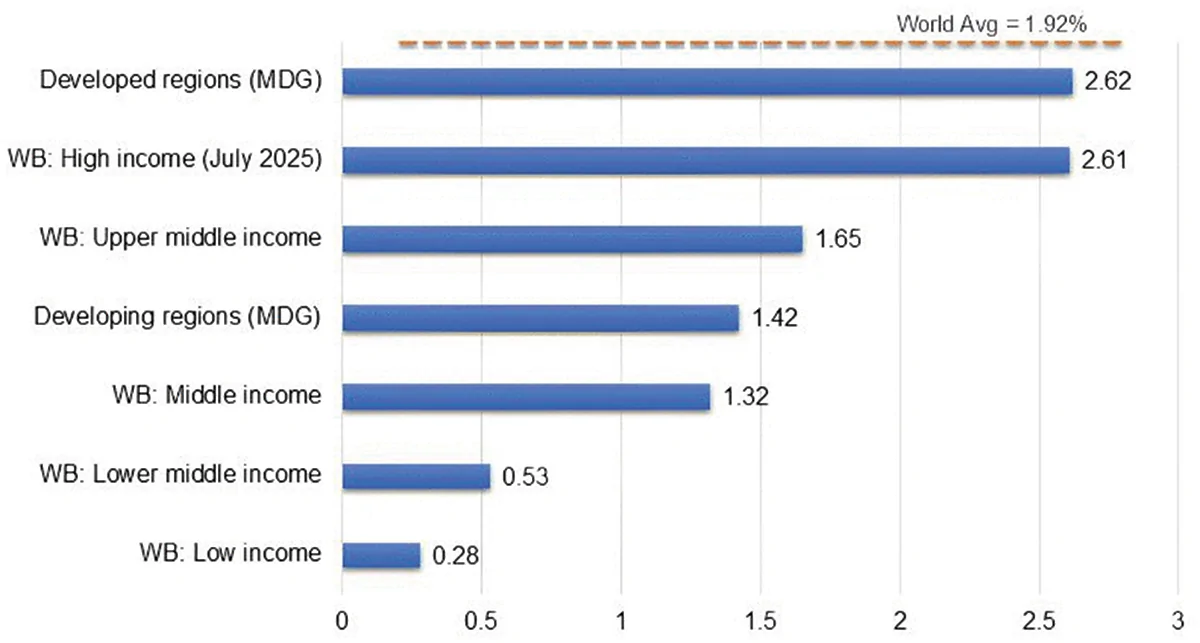
Gross domestic expenditure on R&D as a percentage of GDP, by World Bank income classification (2023). Data are drawn from the UNESCO Institute for Statistics (UIS) 2026 R&D data release and World Bank GERD indicators. The dashed vertical line represents the global average of 1.92%. Bars are sorted in descending order of R&D intensity. High-income countries spend nearly ten times more than low-income countries (2.61% vs. 0.28%), highlighting the structural inequities in global research capacity. Source from https://uis.unesco.org [accessed 30 June 2026].

Despite decades of investment in global health, Africa remains underrepresented in the generation of scientific knowledge needed to address its own health challenges [1–3]. This persistent imbalance reflects not a lack of scientific talent, but decades of under-investment in African-led research systems [4]. Although sub-Saharan Africa bears approximately 26% of the global disease burden [5] it contributes less than 1% of global research output and invests under 1% of gross domestic product (GDP) in research and development [6–8]. To provide a more granular and comparable picture of this disparity, Figure 1 presents regional-level research and development expenditure as a percentage of gross domestic product (GDP), using the most recent data from the UNESCO Institute for Statistics and the World Bank. This figure illustrates that several low- and middle-income countries, including Egypt (~1.0% of GDP), Kenya (~0.8% of GDP), and South Africa (~0.6% of GDP), have made comparatively greater investments in research and development [9]. Although these investments remain below international targets, they demonstrate that sustained domestic financing for research is achievable within the resource constraints faced by many low- and middle-income countries. The consequences of this disparity extend beyond limited scientific productivity to reduced innovation, weakened health systems, and continued reliance on externally driven research agendas that may not fully reflect local priorities [10,11].

These inequalities are reinforced by interconnected structural barriers, including inadequate research funding, limited political prioritization of science and technology, underdeveloped laboratory infrastructure, high cost of scientific equipment and reagents, and the continued migration of skilled researchers to high-income countries [1,12–14]. Collectively, these constraints hinder the development of sustainable research ecosystems capable of generating locally relevant evidence and responding effectively to Africa’s health priorities.

Recognizing these challenges, research capacity-strengthening (RCS) initiatives have increasingly evolved from supporting individual research projects towards strengthening research ecosystems through sustained investments in people, institutions, leadership, and collaborative networks [15,16]. Programmes such as the Wellcome Africa Asia Programme, the European & Developing Countries Clinical Trials Partnership (EDCTP), the Fogarty International Center, the Consortium for Advanced Research Training in Africa (CARTA), and the Carnegie Next Generation of Academics in Africa (NGAA) have demonstrated that long-term investments in mentorship, institutional development, and regional collaboration can strengthen scientific leadership across the continent [17–21]. However, evaluations of these programmes have largely focused on conventional indicators including publications, grant income, and training outputs, with comparatively limited attention given to participants’ perspectives on how such programmes shape scientific independence, leadership development, and long-term career trajectories.

The GSK Africa Open Lab was established within this evolving RCS landscape to strengthen African-led research through an integrated model that combines competitive research funding with mentorship, leadership development, scientific networking, and sustained engagement among early-career researchers. Rather than supporting isolated research projects, the programme seeks to develop a community of scientists capable of leading locally relevant research, strengthening institutional research capacity, and influencing health policy across the continent.

Despite growing evidence of the outputs generated by research capacity-strengthening programmes, less is known about the mechanisms through which participants experience their professional development and contribute to strengthening research ecosystems. Understanding these experiences is essential for designing RCS initiatives that are equitable, sustainable, and responsive to African priorities. This collaborative reflective analysis by members of the inaugural GSK Africa Open Lab cohort examined how participation in the programme influenced research capacity, scientific independence, leadership, and career development, while identifying the programme features that facilitated these outcomes, the structural challenges that persisted, and lessons for the design of future research capacity-strengthening initiatives in Africa. This analysis was guided by a single overarching research question: ‘*How has your participation as part of the inaugural cohort of the GSK AOL programme influenced your research capacity, scientific independence, and career trajectories as an early-career African researchers?’* To address this question, we pursued three objectives: (i) to describe the key features of the GSK Africa Open Lab model; (ii) to document the research, mentorship, and career outcomes of the inaugural cohort; and (iii) to identify lessons to inform the design of future research capacity-strengthening programmes. All 11 members of the inaugural cohort participated in a structured collaborative reflection process conducted between July and October 2025. This process combined facilitated group discussions with a structured written reflection tool covering four domains: research capacity and independence; mentorship and access to networks; institutional and policy impact; and programme limitations. The reflections were analysed using Ritchie and Spencer’s Framework Method [22]. Interpretation was further informed by selected Organisation for Economic Co-operation and Development/Development Assistance Committee (OECD/DAC) evaluation criteria, specifically relevance, effectiveness, sustainability, and coherence [23]. The indicators used to characterize programme outcomes, publications, follow-on funding, career progression, policy engagement, and laboratory capacity development, reflect measures commonly used in evaluations of comparable research capacity-strengthening initiatives, including CARTA, the Wellcome Trust African Institutions Initiative, and Fogarty International Center training programmes. These indicators also emerged inductively from the structured reflection process.

### Our reflexivity statement

All authors are members of the inaugural cohort of the GSK Africa Open Lab and, therefore, have an inherent interest in representing the programme positively. To reduce the risk of this bias shaping the analysis, several measures were built into the reflection process. Reflections were first collected through a structured written instrument before manuscript drafting began. Three authors independently coded the reflections thematically and then compared their interpretations. Any differences were resolved through discussion and consensus with the wider author team. In addition, the manuscript was intentionally structured to present critical reflections and programme limitations alongside reported strengths and achievements.

### Positioning the model among African research capacity-strengthening initiatives

The GSK Africa Open Lab can be understood within the broader landscape of African research capacity-strengthening initiatives, alongside programmes such as the Wellcome Trust’s African Institutions Initiative, the European and Developing Countries Clinical Trials Partnership (EDCTP), the United States National Institutes of Health Fogarty International Center training programmes, the Carnegie Corporation’s Next Generation of African Academics initiative, and the Consortium for Advanced Research Training in Africa (CARTA). Although these programmes share a commitment to strengthening research capacity, the GSK Africa Open Lab appears distinctive in its combination of features: direct principal-investigator-level grant ownership for early-career researchers, structured leadership development, a multi-disease research scope spanning malaria, tuberculosis, antimicrobial resistance, and enteric infections, and an alumni-as-mentors model in which earlier cohorts support those who follow. To our knowledge, this particular combination has not been replicated in the same form elsewhere in the region.

### A model for sustainable research ecosystems

The GSK Africa Open Lab was established as a follow-on initiative to the previous Africa NCD Open Lab [24], to strengthen African-led research capacity through a comprehensive, ecosystem-oriented approach. Through competitive grants of up to £75,000, structured mentorship, and access to technical training, the programme supports early-career scientists working on high-burden diseases including malaria, tuberculosis, antimicrobial resistance, and enteric infections [25].

Launched in 2022, the GSK Africa Open Lab has so far supported two cohorts of researchers, with recruitment for a third cohort currently underway. The inaugural cohort brought together 11 early-career researchers from eight countries across sub-Saharan Africa. Each received funding of up to £75,000 over an initial three-year period, together with structured mentorship and technical training. Building on the success of the inaugural programme, the funding available for subsequent cohorts (Cohorts 2 and 3) was increased up to £100,000 per award. The programme’s sustainability is increasingly being reinforced through an emerging alumni mentorship model, in which members of the inaugural cohort are taking on faculty and co-supervisory roles in later cohorts. In addition, several researchers from the first cohort have gone on to secure follow-on funding from major funders, including the Gates Foundation and the Wellcome Trust, extending the programme’s catalytic influence well beyond the original period of direct support.

Instead of concentrating only on short-term outputs, the initiative places strong emphasis on sustainable, long-term capacity development. By supporting early career researchers as principal investigators, the programme promotes scientific independence and institutional ownership of research agendas. It also fosters collaboration across institutions and disciplines, enabling knowledge exchange and collective problem-solving.

This manuscript draws on the experiences, outputs, and professional trajectories of the inaugural cohort to demonstrate how the GSK Africa Open Lab model strengthens research ecosystems, enhances scientific leadership, and supports sustainable research careers. The programme’s emphasis on mentorship, collaboration, and data stewardship further positions it as a replicable framework for funders and policymakers seeking to strengthen research capacity in low- and middle-income settings.

The GSK Africa Open Lab also sits within a wider ecosystem of international and South–South collaboration in global health research. Brazil, for instance, has a longstanding tradition of public health research dating back to the late nineteenth century, much of it shaped through collaboration with French institutions. Since the 2000s, the Oswaldo Cruz Foundation (FIOCRUZ), including through its office in Mozambique, has extended this legacy through more structured partnerships with African institutions. Similar efforts can be seen in India–Africa research initiatives and regional networks such as the West and Central African Council for Agricultural Research and Development (CORAF). Together, these examples highlight both the value of South–South collaboration and the extent to which it remains insufficiently developed relative to need. In this context, the GSK Africa Open Lab complements existing efforts through an industry-funded, scientist-led model that places particular emphasis on research independence rather than institutional dependency.

## Quantifying impact

### From seed grants to systemic change

The GSK Africa Open Lab initiative demonstrates impact beyond individual-level capacity development, contributing to institutional strengthening and broader research system transformation. Grantees have produced peer-reviewed publications, established independent research programmes, and secured follow-on funding from international agencies, indicating a clear trajectory from early investment to sustained research productivity. One cohort member described the catalytic role of the programme as follows:

*The GSK Africa Open Lab grant was just the beginning. That initial funding opened doors to a major Gates Foundation grant for malaria molecular surveillance in refugee populations across Uganda, South Sudan, and Burundi. More importantly, I recently contributed to Uganda’s Ministry of Health antimalarial drug resistance strategy and the Malaria Elimination Strategic Plan 2026–2030.*

These outcomes illustrate how relatively modest, strategically deployed investments can generate substantial scientific and policy-relevant returns. Beyond individual achievements, the programme has contributed to strengthening institutional research environments through improved research management practices, enhanced laboratory capacity, and the development of mentorship structures. Alumni frequently assume leadership roles, supervise graduate students, and contribute to institutional research agendas thereby extending the programme’s impact beyond the initial funding period. As another cohort member noted, *‘the leadership training provided did not just teach management skills, but equipped me to navigate complex field environments and communicate effectively across diverse teams.’* Collectively, these outcomes demonstrate how targeted investment, when embedded within supportive institutional ecosystems, can foster sustainable research capacity and organizational resilience.

Across the inaugural cohort, these gains are reflected in an expanding body of follow-on funding secured from international agencies, the total value of that funding, graduate students supervised, engagement with government and policy stakeholders, and training activities delivered to junior researchers and technical staff. These indicators were self-reported by cohort members through the structured reflection instrument. In addition, because several projects remain ongoing, the programme’s longer-term downstream effects could not yet be fully assessed and will require future evaluation.

The key expected outputs and impacts of the GSK Africa Open Lab inaugural cohort are summarized in Table 1.

**Table 1.** Characteristics, research focus, and potential outcomes of projects undertaken by the inaugural GlaxoSmithKline Africa Open Lab (GSK-AOL) cohort.

Country	Area of Research	Study Title	Potential Outcomes
Burkina Faso	Malaria Immunology	Placental Malaria: Identification of Host Potential Biomarkers Associated with the Disease	Reveal the immune mechanisms that drive susceptibility or resistance to placental malaria Define host immune signatures associated with placental malaria Uncover the temporal dynamics of maternal immunity during malaria infection in pregnancy Elucidate the relationship between placental inflammation and adverse birth outcomes Establish a systems immunology framework for studying malaria in pregnancy
Gabon	TB Epidemiology and Genomics	Molecular Epidemiology, Strains genotyping of multi-drug-resistant Tuberculosis circulating in Central Africa Region (MESTCAR STUDY)	Identify transmission networks and the geographic distribution of MDR-TB lineages to inform targeted tuberculosis control strategies Detect genetic markers associated with drug resistance to strengthen molecular surveillance, improve diagnostics, and guide individualized treatment Generate regional evidence to support cross-border tuberculosis surveillance, policy development, and antimicrobial resistance control. Strengthen laboratory and genomic surveillance capacity for MDR-TB through regional collaboration and data sharing
Ghana	TB Immunology	Utility of improved techniques of specimen collection by sputum induction and the evaluation of non-sputum-based biomarkers for pediatric tuberculosis diagnosis in Ghana	Improve specimen collection methods in children with tuberculosis Produce validated non-sputum biomarkers that can shorten the time to diagnosis and improve the overall tuberculosis testing experience for children and caregivers Establish induced sputum hubs at study sites for improved testing accuracy Train clinicians and supporting allied health professionals on induced sputum algorithm for children with TB in Ghana
Ghana	Paediatric HIV and TB	Lung function in Ghanaian children with pulmonary Tuberculosis at diagnosis and after treatment: a prospective cohort study	Provide the first Ghanaian longitudinal data on lung function impairment in children following TB treatment Identify risk factors for post-TB lung function impairment, enabling early detection Inform national and international guidelines on monitoring lung health in children post-TB, with direct input into Ghana’s National TB Control Programme
Ghana	Enteric Infections and Genomics	Molecular Epidemiology and Antigenic Characterization of Noroviruses in the Paediatric Population of Ghana	Generate robust estimates of the national pediatric norovirus disease burden and provide critical inputs for cost-effectiveness analyses to support evidence-based vaccine introduction and policy decisions. Inform optimal norovirus vaccination schedules and support modelling of vaccine impact, duration of protection, and population-level herd immunity. Generate the genomic and antigenic data required to design a broadly protective chimeric or multivalent virus-like particle (VLP) norovirus vaccine that confers cross-genogroup and cross-genotype immunity, reducing the need for frequent reformulation in response to viral evolution.
Kenya	Enteric Infection and Antimicrobial Resistance	Epidemiology of Multidrug-resistant *Shigella* in Nairobi, Kenya: The role of asymptomatic carriage and environment	The burden of shigellosis among the communities in urban informal settlements. The role of asymptomatic healthy carriers and the environment in the transmission of Shigella. The burden of multidrug-resistant Shigella in communities residing in urban informal settlements.
Mali	Malaria Vaccines	Evaluating antibody responses longitudinally and correlation to clinical malaria in children who received seasonal vaccination with RTS,S/AS01E with and without seasonal malaria chemoprevention in Mali	Identify immune correlates of protection following RTS,S/AS01E vaccination, providing mechanistic insights into vaccine-induced immunity and sustained protection against clinical malaria. Generate evidence to optimize RTS,S vaccination strategies by informing booster schedules, duration of protection, and integration with seasonal malaria chemoprevention (SMC) in high-burden settings. Provide policy-relevant evidence on RTS,S-mediated protection and its integration with SMC, while strengthening local capacity for malaria vaccine and immunology research.
Nigeria	Malaria Genomics	Assessment of Drug and Diagnostic Resistance Signature in *Plasmodium falciparum*: Implications on Strategies for Malaria Control in Nigeria	Generate Plasmodium falciparum genetic data to support reviews of antimalarial drug and diagnostic policies in Nigeria. Build and transfer genomic capacity for tracking malaria parasites using portable ONT sequencing in Nigeria
Nigeria	Disease Surveillance and Data systems	Epidemiology and Treatment Outcomes of Drug-Resistant Tuberculosis among Children and Adolescents in Nigeria	Identify clinical, demographic, and programmatic factors associated with treatment outcomes to improve patient management and survival Generate evidence to optimize pediatric and adolescent DR-TB diagnosis, treatment, and follow-up within the National Tuberculosis Programme Inform national policies and clinical guidelines to strengthen DR-TB care and improve outcomes among children and adolescents Strengthen the evidence base for resource allocation and implementation of child- and adolescent-centered DR-TB control strategies
Uganda	Malaria Immunology and Genomics	Surveillance of malaria parasitemia, drug and diagnostic resistance in refugee children entering Uganda	Determine the prevalence of *Plasmodium falciparum* and non-*falciparum* malaria among newly arrived refugees to inform malaria screening, treatment, and prevention strategies Map the distribution of genetic markers of antimalarial drug and diagnostic resistance to guide malaria control policies and refugee health programmes Characterize the genomic relatedness and spread of drug- and diagnostic-resistant malaria parasites to improve understanding of resistance emergence and transmission through human migration.
Uganda	Sepsis, Antimicrobial Resistance and Genomics	Detection of neonatal bloodstream infections using next generation sequencing in Uganda	Elucidate the causes of culture-negative neonatal sepsis among critically ill Ugandan newborns Define the bacterial pathogen profile to inform antimicrobial stewardship and implementation of WHO AWaRe-guided antibiotic prescribing Identify diagnostic and prognostic biomarkers, including protein signatures associated with neonatal sepsis Characterize antimicrobial resistance genomic signatures to strengthen the global evidence base on neonatal AMR in low- and middle-income countries Establish a platform for future collaborative neonatal sepsis and antimicrobial resistance research

### The critical role of mentorship and research networks

Mentorship and professional networks emerged as central enabling factors in the success of the cohort. Through structured mentorship, peer learning, and cohort-based engagement, cohort members accessed technical guidance, career development support, and collaborative opportunities that extended beyond the duration of the grant.

Mentorship within the GSK Africa Open Lab is led primarily by African researchers, with additional technical input from GSK scientists and international collaborators. In this way, the programme reflects both North–South and South–South collaboration while maintaining strong leadership from African researchers. The mentorship model is layered, combining technical support from GSK scientists with discipline-specific guidance from external academic mentors, including members of the GSK Africa Open Lab’s scientific advisory board, colleagues from the London School of Hygiene and Tropical Medicine, the University of the Witwatersrand, the University of Ghana, and the Kwame Nkrumah University of Science and Technology. Senior African scientists remain actively involved throughout the programme, helping to ensure that scientific leadership is locally grounded and contextually relevant. Members of the inaugural cohort are also beginning to take on mentoring and faculty roles for subsequent cohorts through an emerging alumni pathway. Further strengthening mentorship led directly by African institutions represents an important opportunity for future iterations of the programme.

Participation in the GSK Africa Open Lab facilitated access to international research networks, advanced training opportunities, and engagement in national and regional research initiatives. One cohort member reflected:

*Growing up in rural Burkina Faso, I never imagined leading immunology research or being nominated for international prizes. The GSK Africa Open Lab didn’t just fund my project on placental malaria biomarkers; it connected me to networks at the London School of Hygiene and Tropical Medicine and opened doors I didn’t know existed.*

These professional networks frequently served as entry points to further funding, policy engagement, and leadership roles. Importantly, the programme’s emphasis on community-building fostered a collaborative rather than competitive research culture. This environment enabled early-career researchers to build confidence, exchange expertise, and develop the professional relationships necessary for sustained engagement in research and policy-relevant work.

### Navigating Open Science and data sovereignty

Open Science is central to advancing equitable and collaborative research, yet its implementation in African contexts requires careful consideration of infrastructure, governance, and data sovereignty [26,27]. The GSK Africa Open Lab promotes the adoption of FAIR (Findable, Accessible, Interoperable, Reusable) data principles while recognizing the need to safeguard local ownership and ethical use of data.

Regional and international frameworks, including the UNESCO Recommendation on Open Science and national science policies, provide guidance for balancing openness with protection of local interests [28,29]. Investments in digital infrastructure, data repositories, and researchers training are essential to operationalizing these principles [30,31]. The programme’s emphasis on responsible data stewardship ensures that research outputs contribute not only to global knowledge but also to local health systems, policy development, and capacity building [30]. By strengthening data governance and promoting equitable access, the GSK Africa Open Lab supports a more inclusive and sustainable model of scientific collaboration.

The programme contributes to data sovereignty principally through capacity strengthening rather than infrastructure development. It supports training in data management, responsible data sharing, and data stewardship, including data management planning, the deposition of datasets in institutional and regional repositories, and advocacy aligned with the UNESCO Recommendation on Open Science. At present, however, the programme does not fund the development of data-centre infrastructure, and investment in this area remains an important future priority for the region.

### Aligning research with regional disease burden

A defining strength of the GSK Africa Open Lab is its alignment with the region’s most pressing health challenges. Supported projects address diseases that contribute disproportionately to morbidity and mortality across sub-Saharan Africa. For example, research on post-tuberculosis lung disease in Ghana is generating evidence to inform national treatment guidelines. As one researcher based at a tertiary teaching hospital explains:

*Ghana’s national TB guidelines have no provisions for long-term follow-up of post-TB lung disease in children, a critical gap currently being documented. A spirometry study among Ghanaian children is generating locally relevant evidence to transform reactive treatment protocols into comprehensive, long-term care pathways.*

Similarly, studies on multidrug-resistant *Shigella* in urban informal settlements are informing antimicrobial stewardship strategies. As noted by a programme-supported researcher:

*Through a GSK-funded study, information on antimicrobial resistance levels is being provided to primary healthcare facilities data that are critical for guiding empiric treatment decisions among clinicians. It is anticipated that this will help reduce inappropriate antibiotic prescriptions and limit the emergence and spread of antimicrobial resistance in urban informal settlements.*

These examples demonstrate how locally led research can directly inform clinical practice and public health policy. By grounding research priorities in the regional disease burden, the programme ensures that scientific outputs are relevant, actionable, and responsive to local health needs.

### Advancing career progression and research independence

The GSK Africa Open Lab has played an important role in supporting career progression and strengthening research independence among early-career scientists. Through a combination of grant ownership, mentorship, and leadership development, participants have developed core skills in project management, grant writing, scientific communication, and team leadership.

Since securing the award, members of the inaugural cohort have collectively supervised numerous graduate students, taken on institutional leadership responsibilities, and secured substantive research positions, providing early quantitative evidence of the programme’s contribution to longer-term career advancement. These evolvements are drawn from the structured reflection instrument, and a more comprehensive assessment of leadership development and research trajectories will be undertaken once ongoing projects have been completed.

Many alumni have progressed from research assistant roles to principal investigator positions, established independent research groups, and secured competitive follow-on funding. For several cohort members, the programme marked a clear turning point by creating an opportunity to lead their own research and mentor more junior colleagues. One cohort member described the grant as transformative because it enabled full ownership of a research project and the chance to support junior researchers. Another emphasized the programme’s role in advancing his scientific autonomy, noting: ‘*This project has allowed me to build regional capacity in molecular diagnostics and to understand the transmission dynamics of multidrug-resistant TB strains*.’ Together, these experiences underscore the value of investing early in research leadership and of providing structured support that enables sustainable scientific careers.

## Programmatic limitations and systemic barriers

Despite its successes, the GSK Africa Open Lab operates within broader structural constraints. Limited opportunities for mid- and senior-career researchers create a gap in the research pipeline, as those who move beyond early-career eligibility often struggle to secure the sustained support needed to consolidate their programmes and lead larger collaborative initiatives.

The outcomes described in this reflective analysis cannot be attributed solely to participation in the programme. Several factors may also have shaped these trajectories, including selection bias in the recruitment of the inaugural cohort, concurrent training and funding opportunities accessed independently of the programme, broader shifts in the global research funding landscape, and social desirability bias in self-reported reflections. We also recognize the possibility of temporal confounding, as the inaugural cohort participated during a period of heightened global investment in African research capacity in the aftermath of the COVID-19 pandemic. This wider context may itself has created opportunities that cohort members were particularly well placed to take advantage of. For this reason, the findings presented here should be understood as reflective insights rather than evidence of direct causality.

Because this manuscript is a reflective analysis rather than a formal programme evaluation, no independent or third-party assessors were involved in data collection or analysis. Future evaluations would, therefore, benefit from incorporating external assessment. Similarly, the indicators used to describe programme outcomes were not specified prospectively; instead, they were informed by established research capacity-strengthening evaluation frameworks and identified inductively through the structured reflection instrument.

We are also unable to comment on GSK’s future institutional commitments beyond what is already publicly available, and we acknowledge this as a further limitation of the present analysis. Broader questions of programme sustainability are, therefore, considered separately in the Conclusion.

Challenges related to equitable access also remain. Although the inaugural cohort included researchers from both Anglophone and Francophone Africa, representation from fragile and conflict-affected settings was limited. This reflects wider logistical, linguistic, and security-related barriers that may unintentionally exclude highly capable scientists working in such contexts, thereby perpetuating existing geographic inequities in African research. In addition, infrastructure constraints, including unreliable electricity, limited laboratory capacity, and inadequate digital systems, continue to impede research productivity and long-term sustainability across many institutions.

The programme’s reliance on external funding also highlights a deeper structural challenge: the continued underinvestment in research and development by many national governments. At the same time, changing patterns in global health financing, including reduced support from traditional donor agencies and competing priorities in high-income countries, may further threaten progress in African research capacity development. Without sustained domestic commitment, gains achieved through externally funded initiatives may prove difficult to maintain over time. Addressing these limitations will require coordinated investment from governments, funders, and research institutions to strengthen the broader ecosystem in which programmes such as the GSK Africa Open Lab operate.

## Lessons learned and implications for future programmes

Several important strengths emerge from the GSK Africa Open Lab experience. These include direct principal-investigator-level grant ownership for early-career researchers, which helps foster genuine scientific independence; a cohort-based model that builds community and sustains peer support and collaboration beyond the funding period; structured leadership development; and a multi-disease focus that has enabled the programme to support work across malaria, tuberculosis, antimicrobial resistance, and enteric infections.

At the same time, the experience also points to areas that would benefit from further strengthening. These include the limited engagement of researchers in fragile and conflict-affected settings, the absence of a structured progression pathway for those moving beyond the early-career stage, uneven parity within some South–South mentorship arrangements, and the lack of a prospective Theory of Change to guide programme design and evaluation from the outset.

Building on these strengths while addressing these gaps, we propose several practical priorities for future iterations of the programme. These include establishing a mid-career bridge funding mechanism to support research leaders beyond early-career eligibility, developing a prospective indicator framework before programme implementation, undertaking a mentorship equity audit to ensure a more balanced mix of South–South and North–South support, and introducing a targeted outreach strategy to improve participation from under-represented settings. Taken together, these lessons underscore the importance of systemic, long-term approaches to research capacity strengthening.

## Conclusion

### Call to action: a blueprint for strategic investment in African research capacity

The experience of the GSK Africa Open Lab shows that, when African researchers are supported through targeted investment and enabling research environments, they are well positioned to produce high-quality, locally relevant science. The central challenge is not a lack of talent or human capacity, but the absence of sustained, coordinated investment frameworks that allow research ecosystems to grow and endure. Strengthening research capacity in Africa, therefore, requires deliberate collaboration among governments, funding agencies, industry partners, and academic and research institutions. Incremental and fragmented support is not enough to overcome the structural inequities that continue to limit locally led scientific innovation. What is needed instead is a more ambitious, coordinated, and transformative approach to building resilient and self-sustaining research ecosystems across the continent.

The recruitment of a third cohort, as confirmed on the GSK Africa Open Lab website, suggests that the programme is continuing beyond its initial phase. Even so, sustainability of this kind cannot depend on a single industry partner, however, committed or well resourced. Lasting impact will require more diversified funding mechanisms, stronger local ownership, and broader engagement from governments, academic institutions, and other funding bodies.

African governments must take primary responsibility for strengthening domestic research systems. Allocating at least 2% of national health budgets to research and development would represent an important step towards reducing dependence on external funding and ensuring national ownership of research priorities. Equally important is the creation of national career development grant schemes for early- and mid-career scientists, which would help secure the next generation of African research leaders and reduce the continued migration of skilled researchers to better-resourced settings. Countries such as Egypt, Kenya, and South Africa already demonstrate that greater investment is possible. Their research and development expenditure exceeds that of many regional peers and offers useful examples for other African countries, even though these levels remain below the 1–2% of GDP target recommended in the African Union’s Science, Technology and Innovation Strategy for Africa.

International funding agencies must also reconsider models that prioritize short-term project outputs over long-term institutional development. Investments over five to ten years are essential if research systems are to develop the infrastructure, workforce, and governance capacity needed for sustained scientific progress. Funding programmes should also be co-designed with African researchers so that investments are aligned with locally defined priorities and support more equitable global research partnerships.

Global pharmaceutical companies and other industry stakeholders likewise have an important role to play in sustainable research development. Expanding and institutionalizing collaborative initiatives modelled on the GSK Africa Open Lab could show how industry partnerships can contribute both to scientific discovery and to long-term regional research capacity. Such investment should extend beyond individual projects to include laboratory infrastructure, data science training, and mechanisms for knowledge transfer that leave durable benefits in place. Companies that benefit from health research conducted in low- and middle-income countries carry both a moral and reputational responsibility to support equitable, long-term investment in local research capacity. At the same time, this responsibility should not rest with industry alone, but be shared across governments, research institutions, and funding partners.

Academic institutions must also help redefine how research success is recognized and rewarded. Promotion and career advancement systems should value policy impact, implementation science, mentorship, and community engagement alongside more traditional publication-based metrics. Recognizing these broader forms of contribution would encourage research that not only advances knowledge, but also informs policy, strengthens health systems, and improves population health outcomes. In this regard, journals and funders should adopt responsible research assessment principles such as those outlined in the San Francisco Declaration on Research Assessment (DORA) and the Leiden Manifesto [32,33]. African universities should revise promotion and tenure criteria to formally recognize mentorship, community engagement, and policy translation, while national research councils should develop funding schemes that actively incentivise implementation science and health systems research.

Taken together, these actions offer a practical and achievable roadmap for strengthening African research ecosystems. Without sustained and coordinated investment, progress in addressing the continent’s disproportionate burden of disease will remain limited. With strategic commitment and genuine partnership, however, Africa can move from being seen primarily as a site of research implementation to being recognized as a global leader in scientific innovation and health research leadership.

The GSK Africa Open Lab provides a compelling example of how such investment can be structured. Its experience points to a wider imperative: strengthening African-led research is not only a matter of equity, but also a scientific necessity. The future of global health innovation will depend on a large part on the extent to which African researchers are empowered to lead, collaborate, and generate knowledge within resilient, well-supported research ecosystems.

## Data Availability

Data sharing is not applicable to this article as no data were created or analysed in this study.
